# Quality Indicators for Drug Dispensing in Community Pharmacies: A Scoping Review Protocol

**DOI:** 10.1111/jep.70333

**Published:** 2025-11-28

**Authors:** Lara Joana Santos Caxico‐Vieira, Elindayane Vieira de Souza, Maria Amélia Joyce da Silva Moura, Rafaella de Oliveira Santos Silva, Alessandra Rezende Mesquita, Tácio de Mendonça Lima, Divaldo Pereira de Lyra

**Affiliations:** ^1^ Graduate Program in Health Sciences, Laboratory of Teaching and Research in Social Pharmacy (LEPFS), Department of Pharmacy Federal University of Sergipe São Cristóvão Sergipe Brazil; ^2^ Department of Pharmacy and Pharmaceutical Administration Fluminense Federal University Niterói Rio de Janeiro Brazil

**Keywords:** community pharmacy services, counseling, health care, quality indicators

## Abstract

**Introduction:**

Assessing health service quality, particularly through indicators, is essential for improvement and accountability. However, the literature lacks a comprehensive analysis of how drug dispensing quality is measured.

**Objective:**

This scoping review protocol aims to identify the validated indicators used to assess the quality of drug dispensing in community pharmacies.

**Methods:**

A scoping review will follow the JBI Reviewer's Manual and PRISMA‐ScR guidelines. An electronic search will be conducted in MEDLINE (PubMed), Embase, Scopus, Web of Science and LILACS, using search terms related to “quality indicators, health care”, “dispensing” and “community pharmacies”, along with their synonyms. Study selection and data extraction will be performed independently by two reviewers, disagreements will be resolved by a third. Findings will be synthesized narratively.

**Conclusion:**

This study will identify and critically analyze quality indicators for drug dispensing in community pharmacies, bridging fragmented practices with evidence‐based standards to guide safer, more effective pharmaceutical care.

## 1 Introduction

Medications are essential for preventing, diagnosing, treating, and managing diseases and health conditions [1]. While they offer significant therapeutic benefits, they also carry risks [2]. Poor access and misuse can lead to serious consequences, including adverse drug events, antimicrobial resistance, therapeutic failure, and poor adherence, contributing to increased global morbidity and mortality [1, 3]. Medication‐related harm is now recognized as a major public health concern [4].

To mitigate these risks, pharmaceutical care, delivered through patient‐centered collaboration and interprofessional teamwork, positions the pharmacist as a key agent in safe and effective medication use [5]. This role is operationalized in drug dispensing, which extends beyond mere distribution to include counseling and clinical interventions to optimize drug therapy. Evidence confirms that such comprehensive, pharmacist‐led dispensing practices improve clinical outcomes, reduce harm, and generate cost‐effective value in healthcare [6, 7].

The term “drug dispensing” is used in hospital and community pharmacy settings, but the nature of the practice differs between these contexts. In hospitals, the dispensing it is primarily a logistical function focused on inventory management and workflow efficiency [8], whereas in community pharmacies, it is inherently clinical, centered on patient counseling [7]. These differences underscore the need for specifics evaluation frameworks.

While a recent review has mapped indicators for hospital‐based drug dispensing [9], no comparable set of validated indicators exists to assess quality in community pharmacy practice [10, 11]. In this context, mapping specific indicators allows for the assessment of the quality of drug dispensing provided to patients in community pharmacies [12, 13]. Therefore, this scoping review aims to identify validated indicators used to assess the quality of drug dispensing in community pharmacy settings.

## 2 Review Question

“What are the validated indicators used to assess the quality of drug dispensing in community pharmacies?”.

## 3 Eligibility Criteria

### 3.1 Problem

Eligible studies will be included if they focus on drug dispensing.

### 3.2 Concept

This review focused on the validated quality indicators.

### 3.3 Context

Will be included studies addressing drug dispensing in community pharmacies.

### 3.4 Type of Sources

This review will include studies focused on the development and validation of drug dispensing indicators, as well as those evaluating dispensing quality using validated indicators. Studies assessing broader pharmacy services, but that report general quality indicators encompassing drug dispensing domains, will also be included. “Indicator validation status” will be assessed only during screening, to maximize sensitivity and avoid missing relevant articles early in the search process.

No restrictions will be applied regarding language or publication year. Studies that do not clearly describe the indicators used will be excluded. Additionally, literature reviews, letters, editorials, comments, books/book chapters, guidelines, conference abstracts/proceedings, and studies without full‐text availability will be excluded.

## 4 Methods

This scoping review will be conducted in accordance with the Joanna Briggs Institute (JBI) Manual for Evidence Synthesis and reported following the PRISMA‐ScR checklist and guidelines [14, 15].

### 4.1 Search Strategy

The search in the literature of articles will be guided by PCCo adapted to PICo, with “P” being the problem (drug dispensing), “C” being the phenomenon of interest (validated quality indicators), and “Co” being the context (community pharmacies). The search strategy will use terms related to “quality indicators, healthcare”, “dispensing”, “community pharmacies” and their combinations. The search strategy will employ both standardized term from the controlled vocabulary of the “National Library of Medicines” through the “Medical Subject Headings (MeSH)” and non‐standard terms to extend the search. A search for studies will be conducted in databases: MEDLINE (PubMed), Embase, Scopus, Web of Science and LILACS (Latin American and Caribbean Health Sciences Literature), without data restriction and regardless of the language (Appendix I). The grey literature will be examined for any relevant supplementary literature. This will involve searching Google Scholar for the first hundred records. References found in the included studies will be evaluated to include any potential studies not yet identified.

### 4.2 Definitions Adopted in This Scoping Review

In this review, drug dispensing is defined as the process by which a pharmacist provides medication to a patient, typically in response to a prescription. This process comprises two key stages: (1) Prescription review: involving verification of the prescription's technical and legal completeness, as well as appropriate medication selection; and (2) Patient‐centered care: focusing on the assessment of individual health needs and the delivery of interventions to support safe and effective medication use, including pharmaceutical counseling. Documentation of this process is recommended to enable evaluation and monitoring of patient health outcomes [5, 16, 17, 18].

In this context, quality indicators are conceptualized according to Lawrence and Olesen (1997) as “measurable elements of practice performance for which there is evidence or consensus that they can be used to assess, and thereby improve, the quality of care provided [19]. Their validation may rely on expert consensus and empirical evidence, for example, to establish face validity (apparent relevance to the concept) or content validity (comprehensive coverage of key domain elements). This approach ensures that indicators are not only measurable, but also clinically meaningful and actionable [20, 21].

### 4.3 Source of Evidence Selection

Studies retrieved from the databases will be imported into Rayyan (QCRI) for duplicate removal. Two reviewers (LJSCV and EVS) will independently screen titles and abstracts to identify potentially eligible studies. Full texts of relevant articles will be assessed for final inclusion based on predefined eligibility criteria. Disagreements will be resolved by a third reviewer (ROSS). If full‐text access is unavailable, corresponding authors will be contacted via email and/or ResearchGate platform (www.researchgate.net).

### 4.4 Data Extraction

Two reviewers (LJSCV and MAJSM) will independently extract data using a preformatted Microsoft® Excel® spreadsheet. Disagreements will be resolved through consensus. In cases of missing or unclear information, corresponding authors will be contacted via email. The following data will be extracted: author, publication year, country, study objective, study design, participants/setting, sample size, number of community pharmacies, number of indicators and domains, indicator classification (structure, process, outcome), indicator development methods, validation methods, key findings and reported limitations or biases. For studies that do not explicitly report study design, two reviewers (ROSS and EVS) will independently classify the design, discrepancies will be resolved by consensus.

### 4.5 Data Analysis

Quality indicators and key aspects of drug dispensing reported in included studies will be synthesized using two conceptual frameworks: Donabedian's Structure‐Process‐Outcome (SPO) model [22] and the pharmaceutical care process outlined by Cipolle, Strand, and Morley (2012) [5]. Indicators will be extracted and classified according to the SPO domains. Where original authors did not classify indicators or where classification was inconsistent with Donabedian's framework, the review team will assign or reclassify them through consensus. Additionally, dispensing‐related indicators will be mapped onto the four core domains of the pharmaceutical care process: (1) patient assessment (situation analysis); (2) care planning (clinical reasoning and intervention); (3) follow‐up and evaluation; and (4) documentation [5]. These domains ensure indicators capture not just operational tasks, but also patient‐centered clinical care.

### 4.6 Agreement Level Evaluation

To assess inter‐rater agreement during article selection, Cohen's kappa statistic (*κ*) will be calculated. Agreement levels will be interpreted as follows: *κ* ≤ 0.10 = no agreement; *κ* 0.11–0.40 = weak agreement; *κ* 0.41–0.75 = moderate to good agreement; *κ* > 0.75 = excellent agreement [23].

## 5 Conclusion

The identification of quality indicators for drug dispensing in community pharmacies will generate critical evidence to bridge the gap between fragmented practices and standardized, evidence‐based evaluation frameworks. By identifying, classifying, and critically analyzing existing indicators, this study will support the development of standardized assessment tools and guide policymakers, practitioners, and researchers in future efforts to enhance the quality of pharmaceutical care.

## Conflicts of Interest

The authors declare no conflicts of interest.

## Supporting information

Appendix I: Search strategy.

## Data Availability

Data sharing is not applicable to this article as no datasets were generated or analysed during the current study. Data will be synthesized using a structured approach, with indicators categorized according to two frameworks: Donabedian's SPO model (Structure, Process, Outcome) and the four core domains of the pharmaceutical care process: patient assessment (situation analysis), care planning (clinical reasoning and intervention), follow‐up and evaluation, and documentation. Findings will be presented narratively alongside standardized evidence tables that summarize key indicator characteristics, including authorship, validation status, SPO classification, and alignment with the pharmaceutical care domains. This integrated analytical approach enables meaningful cross‐study comparison, supports evidence‐informed decision‐making, and clearly identifies critical gaps in the current evidence base, thereby guiding future research on the quality of medication dispensing in community pharmacy practice. This protocol has been registered in the Open Science Framework (OSF) registry (DOI: 10.17605/OSF. IO/GN2EU). Any deviations from or amendments to the protocol will be fully documented and reported in the final scoping review.
