# Supplementary material for: Quality Indicators for Drug Dispensing in Community Pharmacies: A Scoping Review Protocol
Source: J Eval Clin Pract. 2025 Nov 28;31(8):e70333. doi: 10.1111/jep.70333 (PMC12662604; doi:10.1111/jep.70333)
Supplement: Supplementary file 1 — Appendix I: Search strategy. [file JEP-31-0-s001.docx]

**Appendix I: Search strategy**

| **Electronic Bibliographic Databases** | **Search Strategy** |
| --- | --- |
| **MEDLINE**  **(PubMed)** | #1 ("Counseling"[MeSH Terms]) OR ("Dispensing"[Title/Abstract] OR "Counseling"[Title/Abstract] OR "Counselling"[Title/Abstract] OR "Advice"[Title/Abstract])  #2 ("Community Pharmacy Services"[MeSH Terms]) OR ("Community Pharmacy Service"[Title/Abstract] OR "Community Pharmacy Services"[Title/Abstract] OR "Community Pharmacy"[Title/Abstract] OR "Community Pharmacies"[Title/Abstract])  #3 (((((("Quality Indicators, Health Care"[MeSH Terms]) OR ("Quality of Health Care"[MeSH Terms])) OR ("Quality Improvement"[MeSH Terms])) OR ("Quality Assurance, Health Care"[MeSH Terms])) OR ("Process Assessment, Health Care"[MeSH Terms])) OR ("Outcome and Process Assessment, Health Care"[MeSH Terms])) OR ("Quality of Health Care"[Title/Abstract] OR "Health Care Quality"[Title/Abstract] OR "Quality of Care"[Title/Abstract] OR "Care Quality"[Title/Abstract] OR "Quality of Healthcare"[Title/Abstract] OR "Healthcare Quality"[Title/Abstract] OR "Quality Indicator*"[Title/Abstract] OR "Quality Improvement*"[Title/Abstract] OR "Quality Assurance*"[Title/Abstract] OR "Quality Assessment*"[Title/Abstract] OR "Quality Management"[Title/Abstract] OR "Process Measure*"[Title/Abstract] OR "Process Assessment*"[Title/Abstract] OR "Quality Norm*"[Title/Abstract] OR "Quality Criteria"[Title/Abstract] OR "Quality Standard*"[Title/Abstract] OR "Quality Metric*"[Title/Abstract] OR "Quality Measure*"OR "Quality Measurement*"[Title/Abstract] OR "Process Criteria"[Title/Abstract] OR "Process Standard*"[Title/Abstract] OR "Process Indicator*"[Title/Abstract] OR "Process Metric*"[Title/Abstract] OR "Process Measurement*"[Title/Abstract] OR "Process Improvement*"[Title/Abstract] OR "Performance Criteria"[Title/Abstract] OR "Performance Standard*"[Title/Abstract] OR "Performance Indicator*"[Title/Abstract] OR "Performance Metric*"[Title/Abstract] OR "Performance Measure*"[Title/Abstract] OR "Performance Measurement*"[Title/Abstract] OR "Performance Assessment*"[Title/Abstract] OR "Performance Improvement*"[Title/Abstract] OR "Indicator*"[Title/Abstract] OR "Quality Indicator* Healthcare"[Title/Abstract] OR "Healthcare Quality Indicator*"[Title/Abstract] OR "Indicator* Healthcare Quality"[Title/Abstract] OR "Health Metric*"[Title/Abstract] OR "Metric*"[Title/Abstract] OR  "Measure*"[Title/Abstract] OR "Measurement*"[Title/Abstract] OR "Outcome* |

|  | Assessment* Health Care"[Title/Abstract] OR "Outcome Assessment*"[Title/Abstract] OR "Assessment*, Outcome*"[Title/Abstract] OR "Outcome* Measure*"[Title/Abstract] OR "Measure*, Outcome*"[Title/Abstract] OR "Outcomes Research"[Title/Abstract] OR "Research, Outcomes"[Title/Abstract] OR "Outcome Stud*"[Title/Abstract] OR "Stud*, Outcome"[Title/Abstract])  #1 AND #2 AND #3 |
| --- | --- |
| **LILACS** | ("Dispensing" OR "Counseling" OR "Counselling" OR "Advice") AND ("Community Pharmacy Service" OR "Community Pharmacy Services" OR "Community Pharmacy" OR "Community Pharmacies") AND ("Quality of Health Care" OR "Health Care Quality" OR "Quality of Care" OR "Care Quality" OR "Quality of Healthcare" OR "Healthcare Quality" OR "Quality Indicator" OR "Quality Indicators" OR "Quality Improvement" OR "Quality Improvements" OR "Quality Assurance" OR "Quality Assurances" OR "Quality Assessment" OR "Quality Assessments" OR "Quality Management" OR "Process Measure" OR "Process Measures" OR "Process Assessment" OR "Process Assessments" OR "Quality Norm" OR "Quality Norms" OR "Quality Criteria" OR "Quality Standard" OR "Quality Standards" OR "Quality Metric" OR "Quality Metrics" OR "Quality Measure" OR "Quality Measure" OR "Quality Measurement" OR "Quality Measurements" OR "Process Criteria" OR "Process Standard" OR "Process Standards" OR "Process Indicator" OR "Process Indicator" OR "Process Metric" OR "Process Metrics" OR "Process Measurement" OR "Process Measurements" OR "Process Improvement" OR "Process Improvements" OR "Performance Criteria" OR "Performance Standard" OR "Performance Standards" OR "Performance Indicator" OR "Performance Indicators" OR "Performance Metric" OR "Performance Metrics" OR "Performance Measure" OR "Performance Measures" OR "Performance Measurement" OR "Performance Measurements" OR "Performance Assessment" OR "Performance Assessments" OR "Performance Improvement" OR "Performance Improvements" OR "Indicator" OR "Indicators" OR "Quality Indicators Healthcare" OR "Healthcare Quality Indicator" OR "Healthcare Quality Indicators" OR "Indicators Healthcare Quality" OR "Health Metric" OR "Health Metrics" OR "Metric" OR "Metrics" OR "Measure" OR "Measurement" OR "Measurements" OR "Outcomes Assessment" OR "Outcomes Assessments" OR "Outcome Assessments" OR "Outcome Assessment" OR "Outcome Measures" OR "Outcome Measure" OR "Outcomes  Research" OR "Outcome Studies" OR "Outcome Study") AND db:("LILACS") |
| **SCOPUS** | ( TITLE-ABS-KEY ( "Dispensing" OR "Counseling" OR "Counselling" OR "Advice" ) ) AND ( TITLE-ABS-KEY ( "Community Pharmacy Service" OR "Community Pharmacy Services" OR "Community Pharmacy" OR "Community  Pharmacies" ) ) AND ( TITLE-ABS-KEY ( "Quality of Health Care" OR "Health |

|  | Care Quality" OR "Quality of Care" OR "Care Quality" OR "Quality of Healthcare" OR "Healthcare Quality" OR "Quality Indicator" OR "Quality Indicators" OR "Quality Improvement" OR "Quality Improvements" OR "Quality Assurance" OR "Quality Assurances" OR "Quality Assessment" OR "Quality Assessments" OR "Quality Management" OR "Process Measure" OR "Process Measures" OR "Process Assessment" OR "Process Assessments" OR "Quality Norm" OR "Quality Norms" OR "Quality Criteria" OR "Quality Standard" OR "Quality Standards" OR "Quality Metric" OR "Quality Metrics" OR "Quality Measure" OR "Quality Measure" OR "Quality Measurement" OR "Quality Measurements" OR "Process Criteria" OR "Process Standard" OR "Process Standards" OR "Process Indicator" OR "Process Indicator" OR "Process Metric" OR "Process Metrics" OR "Process Measurement" OR "Process Measurements" OR "Process Improvement" OR "Process Improvements" OR "Performance Criteria" OR "Performance Standard" OR "Performance Standards" OR "Performance Indicator" OR "Performance Indicators" OR "Performance Metric" OR "Performance Metrics" OR "Performance Measure" OR "Performance Measures" OR "Performance Measurement" OR "Performance Measurements" OR "Performance Assessment" OR "Performance Assessments" OR "Performance Improvement" OR "Performance Improvements" OR "Indicator" OR "Indicators" OR "Quality Indicators Healthcare" OR "Healthcare Quality Indicator" OR "Healthcare Quality Indicators" OR "Indicators Healthcare Quality" OR "Health Metric" OR "Health Metrics" OR "Metric" OR "Metrics" OR "Measure" OR "Measurement" OR "Measurements" OR "Outcomes Assessment" OR "Outcomes Assessments" OR "Outcome Assessments" OR "Outcome Assessment" OR "Outcome Measures" OR "Outcome Measure" OR "Outcomes  Research" OR "Outcome Studies" OR "Outcome Study" ) ) |
| --- | --- |
| **Web of science** | #1 TS=("Dispensing" OR "Counseling" OR "Counselling" OR "Advice")  #2 TS=("Community Pharmacy Service" OR "Community Pharmacy Services" OR "Community Pharmacy" OR "Community Pharmacies")  #3 TS=(“Quality of Health Care” OR “Health Care Quality” OR “Quality of Care” OR “Care Quality” OR “Quality of Healthcare” OR “Healthcare Quality” OR “Quality Indicator” OR “Quality Indicators” OR “Quality Improvement” OR “Quality Assurance” OR “Quality Assessment” OR “Quality Management” OR “Process Measure” OR “Process Assessment” OR “Quality Norm” OR “Quality Criteria” OR “Quality Standard” OR “Quality Metric” OR “Quality Measure” OR “Quality Measurement” OR “Quality Measurements” OR “Process Criteria” OR “Process Standard” OR “Process Indicator” OR “Process Metric” OR “Process Measurement” OR “Process Improvement” OR “Performance Criteria” OR “Performance Standard” OR “Performance Indicator” OR “Performance  Indicators” OR “Performance Metric” OR “Performance Measure” OR |

|  | “Performance Measurement” OR “Performance Assessment” OR “Performance Improvement” OR “Indicator” OR “Indicators” OR “Quality Indicators Healthcare” OR “Healthcare Quality Indicator” OR “Indicators Healthcare Quality” OR “Health Metric” OR “Metric” OR “Measure” OR “Measurement” OR “Outcomes Assessment” OR “Outcome Assessment” OR “Outcome Measure” OR “Outcomes Research” OR “Outcome Studies”)  #1 AND #2 AND #3 |
| --- | --- |
| **Embase** | #1 'dispensing':ab,ti OR 'counseling':ab,ti OR 'counselling':ab,ti OR 'advice':ab,ti #2 'community pharmacy service':ab,ti OR 'community pharmacy services':ab,ti OR 'community pharmacy':ab,ti OR 'community pharmacies':ab,ti  #3 'quality of health care':ab,ti OR 'health care quality':ab,ti OR 'quality of care':ab,ti OR 'care quality':ab,ti OR 'quality of healthcare':ab,ti OR 'healthcare quality':ab,ti OR 'quality indicator':ab,ti OR 'quality indicators':ab,ti OR 'quality improvement':ab,ti OR 'quality improvements':ab,ti OR 'quality assurance':ab,ti OR 'quality assurances':ab,ti OR 'quality assessment':ab,ti OR 'quality assessments':ab,ti OR 'quality management':ab,ti OR 'process measure':ab,ti OR 'process measures':ab,ti OR 'process assessment':ab,ti OR 'process assessments':ab,ti OR 'quality norm':ab,ti OR 'quality norms':ab,ti OR 'quality criteria':ab,ti OR 'quality standard':ab,ti OR 'quality standards':ab,ti OR 'quality metric':ab,ti OR 'quality metrics':ab,ti OR 'quality measure':ab,ti OR 'quality measurement':ab,ti OR 'quality measurements':ab,ti OR 'process criteria':ab,ti OR 'process standard':ab,ti OR 'process standards':ab,ti OR 'process indicator':ab,ti OR 'process metric':ab,ti OR 'process metrics':ab,ti OR 'process measurement':ab,ti OR 'process measurements':ab,ti OR 'process improvement':ab,ti OR 'process improvements':ab,ti OR 'performance criteria':ab,ti OR 'performance standard':ab,ti OR 'performance standards':ab,ti OR 'performance indicator':ab,ti OR 'performance indicators':ab,ti OR 'performance metric':ab,ti OR 'performance metrics':ab,ti OR 'performance measure':ab,ti OR 'performance measures':ab,ti OR 'performance measurement':ab,ti OR 'performance measurements':ab,ti OR 'performance assessment':ab,ti OR 'performance assessments':ab,ti OR 'performance improvement':ab,ti OR 'performance improvements':ab,ti OR 'indicator':ab,ti OR 'indicators':ab,ti OR 'quality indicators healthcare':ab,ti OR 'healthcare quality indicator':ab,ti OR 'healthcare quality indicators':ab,ti OR 'indicators healthcare quality':ab,ti OR 'health metric':ab,ti OR 'health metrics':ab,ti OR 'metric':ab,ti OR 'metrics':ab,ti OR 'measure':ab,ti OR 'measurement':ab,ti OR 'measurements':ab,ti OR 'outcomes assessment':ab,ti OR 'outcomes assessments':ab,ti OR 'outcome assessments':ab,ti OR 'outcome assessment':ab,ti OR 'outcome measures':ab,ti OR 'outcome measure':ab,ti OR  'outcomes research':ab,ti OR 'outcome studies':ab,ti OR 'outcome study':ab,ti |

|  | #4 [embase]/lim NOT ([embase]/lim AND [medline]/lim) #5 #1 AND #2 AND #3 AND #4 |
| --- | --- |
| **GOOGLE SCHOLAR** | ("Counseling" OR "Dispensing" OR "Counselling" OR "Advice") AND ("Pharmacy" OR "Pharmacies") AND ("Quality" OR "Indicator" OR "Indicators" OR “Metric” OR “Metrics” OR “Measure” OR “Measures” OR “Measurement” OR  “Measurements”) |
